# Pathogenesis of Chemical Leukoderma and Chemical‐Induced Vitiligo

**DOI:** 10.1111/1346-8138.70060

**Published:** 2025-11-18

**Authors:** Yasutaka Kuroda, Lingli Yang, Ichiro Katayama

**Affiliations:** ^1^ Department of Pigmentation Research and Therapeutics, Graduate School of Medicine Osaka Metropolitan University Osaka Japan; ^2^ Cosmetic Products Research, Research and Development Kao Corporation Odawara Japan

**Keywords:** animal model, leukoderma, pigmentation disorders, Rhododendrol, vitiligo

## Abstract

Leukoderma/vitiligo is a serious pigmentary disorder that notably impairs the patient's quality of life. In particular, chemical leukoderma (CL)/vitiligo refers to acquired depigmentation of the skin induced by exposure to certain chemicals. In this review, the term “CL” is defined as the temporary, localized loss of pigmentation at the site of direct chemical exposure. When the causative chemical is removed, these skin patches undergo re‐pigmentation, indicating the restoration of melanocytes to their original condition. However, when the chemical‐induced skin depigmentation does not recover after the chemical is removed, or when de novo depigmented lesions emerge, it is classified as chemical‐induced vitiligo. This condition indicates that, even after chemical removal, the mature melanocytes cannot recover because of factors including, but not limited to, autoimmunity, stem cell depletion, and unknown factors. In this review, we summarized the latest pathological findings for each condition, focusing on rhododendrol, which is known to induce both phenotypes and cause an outbreak, which affected nearly 20,000 patients in Japan and other Asian countries.

## 1 Introduction

**FIGURE 1:**
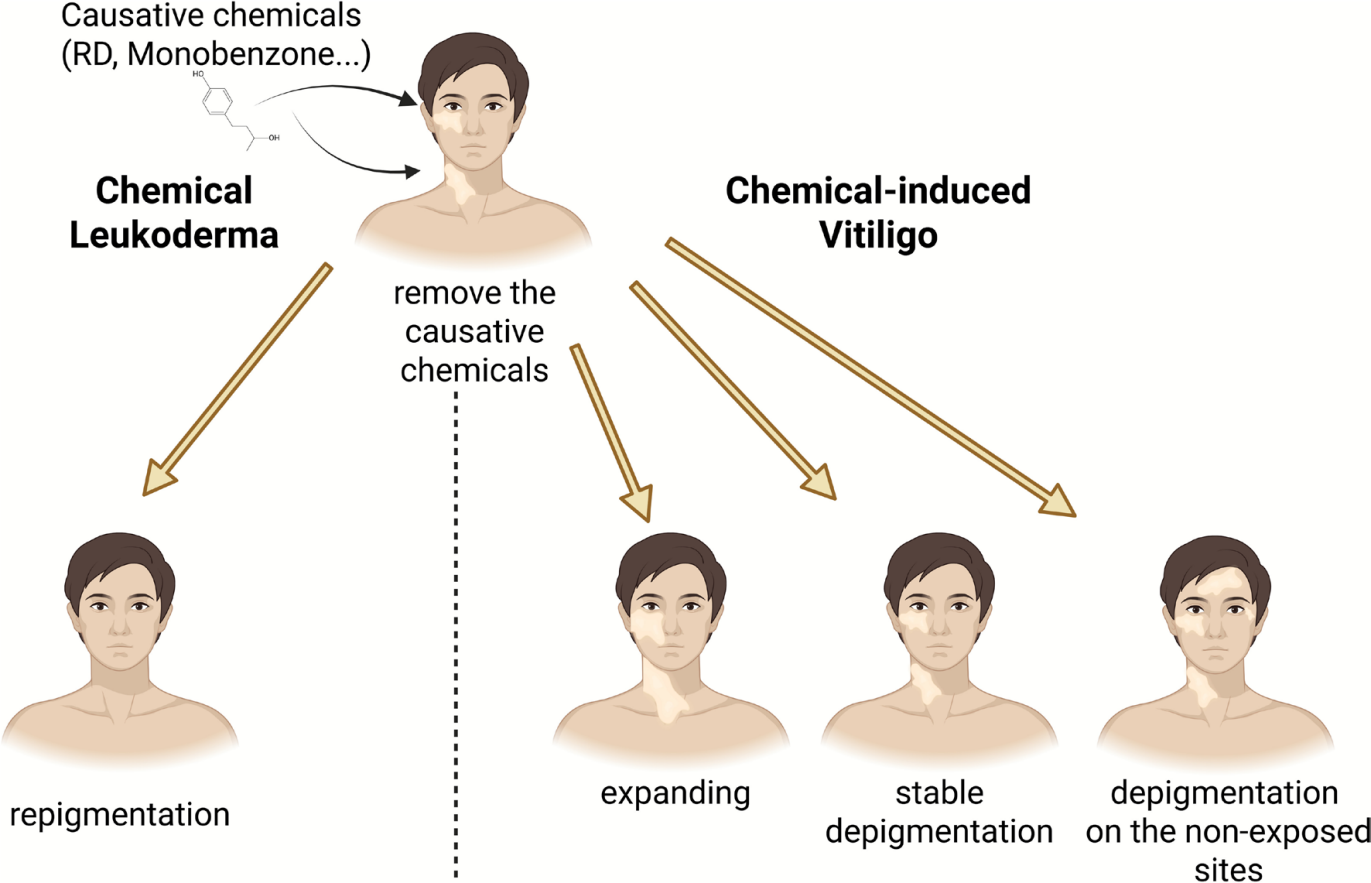
Definition of chemical leukoderma (CL) and chemical‐induced vitiligo (CIV). Following the elimination of the causative chemical, CL undergoes a process of repigmentation and subsequent recovery. In contrast, in the case of CIV, the depigmented area expands, persists, or appears in non‐exposed areas, even after the causative chemical has been removed.

Leukoderma/vitiligo is a serious pigmentary disorder that significantly affects the patient's quality of life. Among pigmentary disorders of the skin, chemical leukoderma (CL)/vitiligo is characterized by depigmentation patches following an exposure to certain chemicals [1, 2, 3]. The term “CL/vitiligo” is also used interchangeably with “contact leukoderma/vitiligo” or “occupational leukoderma/vitiligo,” depending on the route and site of chemical exposure [4]. CL/vitiligo had been frequently reported in occupational contexts, such as rubber glove exposure [5]. However, in recent years, cases of CL/vitiligo caused by household products have increased in India; numerous cases have also been reported in Japan, owing to exposure to skin‐brightening cosmetics [1, 6]. While this condition frequently manifests at the cutaneous site of exposure, approximately 26% of patients in India and roughly 4% of patients exposed to skin‐brightening cosmetics in Japan developed white patches at distant sites not related to the application area. In this review, from a pathological perspective, the term “CL” is defined as temporary depigmentation at the cutaneous site (skin patches) exposed to certain chemicals (Figure 1). Nonetheless, once the chemical is removed, these patches undergo re‐pigmentation, which indicates the restoration of melanocytes to their original condition. However, when the chemical‐induced skin depigmentation does not recover after chemical removal, or when de novo depigmented lesions emerge, it is classified as chemical‐induced vitiligo (CIV) (Figure 1). This condition indicates that, even after the causative chemical is removed, the mature melanocytes cannot return because of factors including, but not limited to, autoimmunity, stem cell depletion, and unknown factors. This review summarizes the latest pathological findings for CL and CIV, focusing on rhododendrol (RD), which is known to induce both phenotypes and cause an outbreak that affected nearly 20,000 patients in Japan and other Asian countries [6, 7].

## 2 Common Pathologies of CL and CIV

**FIGURE 2:**
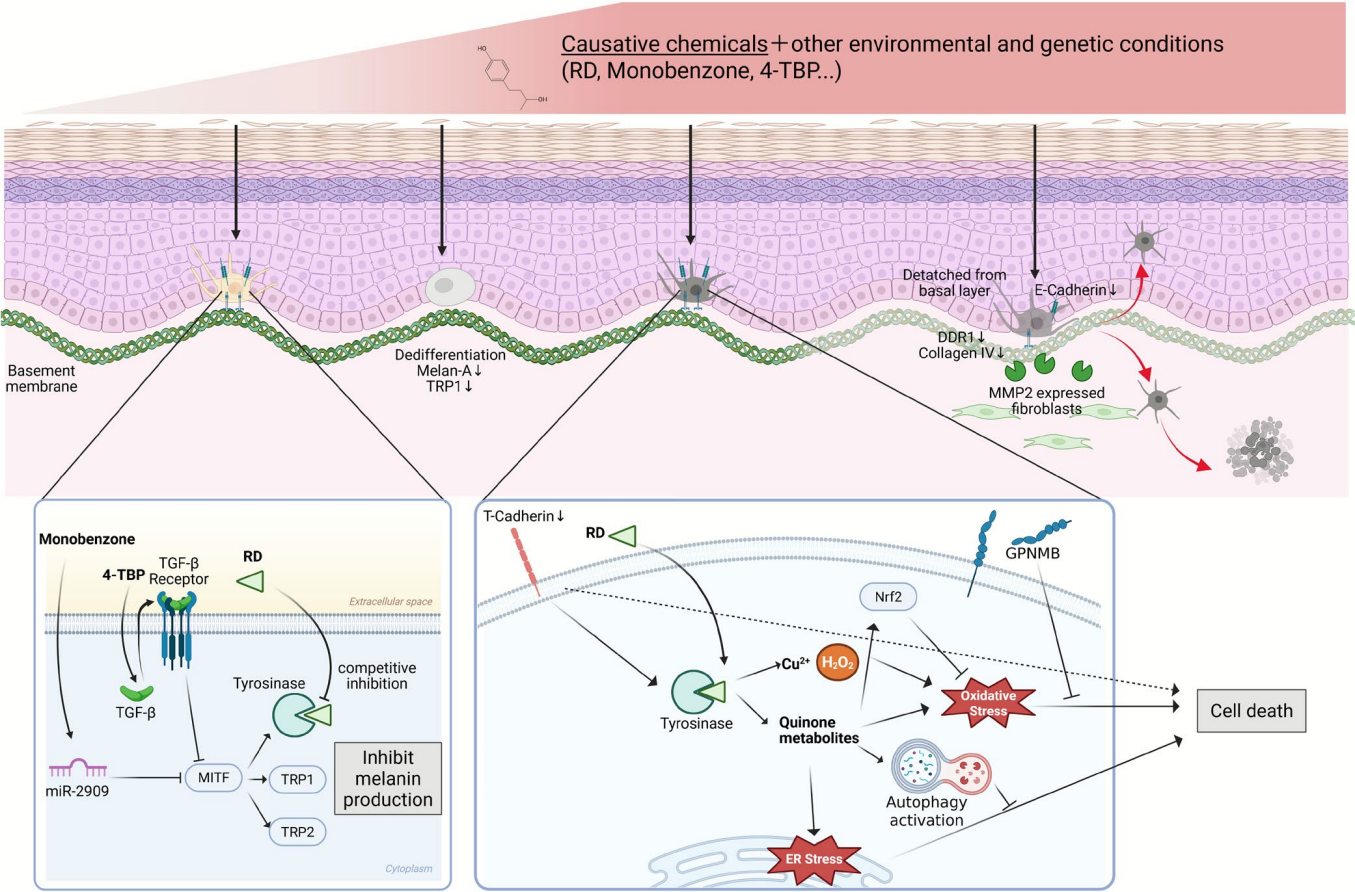
Proposed common mechanisms of chemical leukoderma (CL) and chemical‐induced vitiligo (CIV). The process of depigmentation is induced by a combination of causative chemicals, other environmental factors, and genetic background. Exposure to causative chemicals at levels lower than this threshold can lead to reduced melanin production, resulting in depigmentation. Monobenzone suppresses MITF via miR‐2909, while 4TBP suppresses MITF via TGF‐β signaling, negatively regulating the expression of melanogenesis‐related proteins. RD competitively inhibits tyrosinase, suppressing melanin production. When exposure to causative chemicals exceeds a certain threshold, melanocyte dedifferentiation, cell death, and relocation occur. Melanocyte cell death induced by RD is triggered by the metabolism of tyrosinase, which produces hydrogen peroxide and RD quinone metabolites. These products induce endoplasmic reticulum (ER) stress and oxidative stress. When the amount of stress exceeds the capacity of the intrinsic inhibitory systems, the ultimate outcome is cell death. RD also induces MMP2‐positive fibroblasts in the dermis, causing basement membrane abnormalities. Melanocytes that adhere to the basement membrane detach and relocate toward the stratum corneum or the superficial dermis. This results in the disappearance of melanocytes.

CL and CIV develop when pigment cells or the area surrounding them is directly damaged by causative chemicals. Both conditions are often induced by phenol or catechol derivatives [4]. In particular, *p*‐substituted phenols such as monobenzyl ether of hydroquinone (monobenzone), 4‐tert‐butylphenol (4TBP), and RD induce CL/CIV [5, 6, 8, 9]. These causative chemicals reduce the viability or impair the melanogenic function of melanocytes, which produce the melanin pigment, and induce CL/CIV when genetic and other complex environmental conditions are met (Figure 2). Additionally, changes in the melanocyte niche or the dedifferentiation of melanocytes reportedly contribute to CL/CIV development.

### 2.1 Melanocyte Death

The process of metabolizing RD into catechol produces a quinone compound, which is of greater significance [7, 10]. When tyrosinase metabolizes RD, various quinones are produced in both in vitro and in vivo conditions. These quinones impair melanocytes by inducing endoplasmic reticulum (ER) stress and oxidative stress [11, 12, 13]. The dependence of tyrosinase on cell death has been demonstrated in studies using cell lines with reduced tyrosinase expression or albino mice [12, 13, 14]. Apoptosis is the mode of cell death caused by RD in vitro [13, 15]. Additionally, RD appears to function as a suicide substrate for tyrosinase in vitro, and nonapoptotic cell death may be induced by hydroxyl radicals generated by copper ions and hydrogen peroxide produced during tyrosinase inactivation [16]. In animal models, RD application has replicated CL, but not CIV, in the dorsal regions of guinea pigs and hairless hk14‐stem cell factor (SCF)‐transgenic mice and in the tails of mice and zebrafish. In all these cases, mature melanocytes have declined [12, 17, 18, 19, 20]. However, although tyrosinase‐dependent accumulation of ER stress is reportedly a pathway of melanocyte cell death caused by RD in vivo, no evidence exists that all of the mechanisms detected in vitro, such as apoptosis and oxidative stress, are occurring. As reported by Vidhya et al., monobenzone triggered necrosis, and 4TBP induced apoptosis in both in vitro and ex vivo settings [21]. The pattern of cell death differs markedly depending on the experimental system and chemical inducers. However, under in vitro conditions, the cells are possibly overexposed to chemicals, resulting in effects that would not occur in vivo and in humans. Subsequent research in vivo or ex vivo is imperative to deepen our understanding of these mechanisms.

Conversely, some individuals who are exposed to the causative chemicals but have healthy skin melanocytes, which possess intrinsic detoxification mechanisms, exhibit a non‐universal CL/CIV manifestation. Increasing the intracellular glutathione pools and activating the NRF2 system, NAD(P)H dehydrogenase, quinone 1 (NQO1), and the autophagy pathway with rapamycin can reduce RD toxicity [15, 22, 23]. Genome‐wide association studies have identified *CDH13*/T‐cadherin as a susceptibility gene for RD‐induced leukoderma/vitiligo [24]. In addition, CDH13 knockdown regulates the expression of tyrosinase and apoptosis related molecules, suggesting its ability to enhance RD toxicity. Glycoprotein nonmetastatic melanoma protein B (GPNMB) is a membrane protein that is highly expressed in melanocytes and detected in healthy basal keratinocytes, but not vitiligous keratinocytes [25]. The extracellular domain of GPNMB is released as a soluble form, protecting melanocytes from RD‐ and oxidative stress‐induced toxicity; however, keratinocyte GPNMB is absent in the epidermal basal layer lesions of patients with RD‐induced vitiligo [26]. Therefore, keratinocyte GPNMB may be potentially involved in the pathogenesis of RD‐induced leukoderma/vitiligo. Consequently, the disruption of these detoxification mechanisms may enhance the toxicity of RD and causative chemicals in melanocytes.

### 2.2 Melanogenesis Inhibition

In vitro studies have demonstrated that RD competitively inhibits tyrosinase and reduces melanogenesis [13]. At concentrations that do not cause cell damage, RD downregulates melanogenic proteins in melanocytes and further activates autophagy, thereby reducing melanin production [15]. Moreover, monobenzone functions as a melanogenesis inhibitor via the action of miR‐2909 [27]. Also, 4TBP contributes to melanogenesis reduction by enhancing TGF‐β expression. Patients with vitiligo display increased miR‐2909 expression in the lesional and peri‐lesional skin, suggesting a similar mechanism of melanogenesis reduction in vitiligo. Melanogenesis suppression and melanocyte cell death induction are determined by the chemical's concentration, and cell death might occur when a certain threshold is exceeded.

### 2.3 Melanocyte Niche Destruction

While RD has been demonstrated to induce melanocyte cell death in vitro, evidence indicating cell death occurrence in vivo remains scarce. This paucity may be attributed to melanocytes' potential to undergo changes in their surrounding environment, rather than undergoing cell death. In the hairless hk14‐SCF‐transgenic mouse model, RD application resulted in melanocyte detachment from the epidermal basal layer, accompanied with a temporary decrease in DDR1 and E‐cadherin expression throughout the epidermis. This finding suggests that melanocytes failed to adhere to the basement membrane and the surrounding keratinocytes [28]. In the guinea pig model, RD application did not induce TUNEL‐positive apoptotic melanocytes in the basal layer [29]. Instead, it resulted in the detachment of melanocytes from the epidermal basement membrane, migrating toward either the spinous layer of the epidermis or the dermis. At this time, MMP2 expression is increased in epidermal keratinocytes and dermal fibroblasts, while the expression of type IV collagen, a component of the basement membrane, is decreased. Therefore, melanocyte loss may have been caused by the absence of the niche, which functions as an adhesion scaffold for melanocytes in the epidermal basal layer. A similar pathophysiological mechanism has been observed in vitiligo and RD‐induced vitiligo cases [30] (unpublished data, Professor Katayama, Department of Pigmentation Research and Therapeutics, Osaka Metropolitan University, Osaka, Japan). Dermal melanophages are a common finding in RD‐induced leukoderma and vitiligo. These melanophages appear as dead cells detached from the basement membrane that have dropped off to the dermis and been phagocytosed by macrophages [31]. Currently, the formation and maintenance of melanocyte niches remain largely unclear; thus, further research is needed.

### 2.4 Melanocyte Dedifferentiation

RD induces melanocyte dedifferentiation both in vitro and in vivo, leading to the disappearance of melanocytes expressing mature melanocyte markers, such as melan‐A and TRP1 [32]. Consequently, depigmented patches are formed. In vitiligo, the dedifferentiated melanocyte is induced by a niche that exhibits a characteristic laminin expression pattern in the basement membrane of the epidermis. Dedifferentiated melanocytes have also been confirmed in the ex vivo epidermis of patients with vitiligo, and JNK inhibitors, acting as redifferentiation agents, increase the number of mature epidermal melanocytes. Similar pathological changes might occur in CL/CIV cases; however, further research is warranted for verification.

## 3 Specific Pathologies of CIV

**FIGURE 3:**
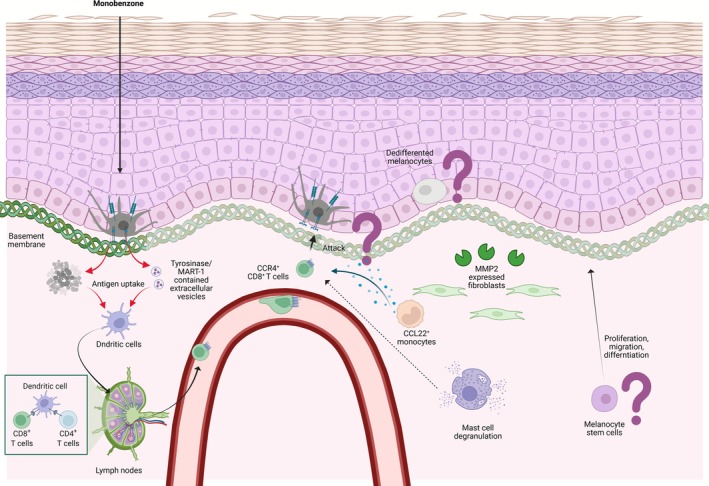
Proposed specific mechanisms of chemical‐induced vitiligo (CIV). In CIV, in addition to mechanisms shared with CL, the induction of autoimmune mechanisms is proposed. Dead melanocytes or extracellular vesicles are phagocytosed by antigen‐presenting cells, leading to the proliferation of CD8‐positive T cells reacting to melanocyte antigens in lymph nodes. CCR4‐positive CD8 T cells, which home to the skin, may migrate to the skin via CCL22 from monocytes and activate by an unknown factor from mast cells and attack melanocytes. Furthermore, the persistent dedifferentiation of melanocytes or the continuous presence of basement membrane abnormalities may impede the process of mature melanocyte settlement. Melanocyte stem cells, which are the source of melanocytes, may be depleted or unable to proliferate, migrate, or differentiate.

In CL, skin pigmentation restoration to healthy levels is achieved through various mechanisms. These mechanisms include the proliferation, differentiation, and migration of melanocytes from stem cells, the migration of melanocytes from the peri‐lesional area, and the reversion of temporarily damaged melanocytes to their original state. In contrast to vitiligo, RD‐induced leukoderma did not involve degeneration of the ER or mitochondria outside the melanosomes [31]. Furthermore, more melanocytes remain in RD‐induced leukoderma lesions than in vitiligo lesions, thereby reflecting its reversible nature. In addition to common pathology, CIV has systemic and continuous effects, maintaining and expanding the pathological state even after the removal of causative chemicals. The most extensively studied hypothesis involves an autoimmune pathway, similar to that for vitiligo (Figure 3). However, a considerable number of cases do not achieve complete remission with immunosuppressants alone, necessitating consideration of other hypotheses (Figure 3).

### 3.1 Autoimmunity to Melanocytes

Depigmented patches may also appear in areas not exposed to the causative chemical, suggesting the potential induction of autoimmunity against melanocytes. This autoimmunity may involve the production of autoantibodies reacting to self‐antigens or the participation of self‐reactive T cells reacting to self‐antigens, concurrent with a decrease in regulatory T cell function. The involvement of self‐reactive CD8^+^ T cells, resident memory T cells, and regulatory T cells has been frequently reported in vitiligo cases [33, 34, 35]. Furthermore, thyroid‐specific autoantibodies and anti‐TYRP2 antibodies were detected in the serum of patients with vitiligo but not in the serum of those with RD‐induced vitiligo [36]. Thyroid‐specific autoantibodies have also been reported to be absent in RD‐induced vitiligo cases. Considering the presence of autoreactive CD8^+^ T cells, while the number of cases is limited, most patients with RD‐induced vitiligo who possess melan‐A‐specific cytotoxic T cells exhibited persistently complete depigmented patches for over 1 year following RD exposure discontinuation [37]. Additionally, patients with RD‐induced vitiligo displayed a substantial presence of CCR4‐positive CD8^+^ T cells within the peripheral blood mononuclear cell population [38]. With prolonged periods of RD exposure discontinuation, the proportion of these cells decreased. The proportion of CCR4‐positive cells among CD8^+^ T cells was significantly higher in the skin of patients with RD‐induced vitiligo; monocytes expressing the cognate ligand CCL22 were also detected in the dermis. Two studies examined HLA types that are critical for epitope recognition. One identified that patients with RD‐induced vitiligo frequently carried HLA‐A*02:01:01 and A*24:02, though this frequency did not differ significantly from that observed in the Japanese population [37]. The other study reported that although the sample size is limited, a notably higher proportion of patients were found to be positive for HLA‐DR4, a risk factor for RD‐induced vitiligo [39]. In patients with RD‐induced vitiligo, degranulated mast cells accumulate in the dermis, a finding similar to that observed in those with vitiligo [40, 41]. Mast cell degranulation is even more frequent in aggravated cases of RD‐induced vitiligo [40]. While the precise role of these mast cells in RD‐induced vitiligo pathogenesis remains poorly understood, mast cells in RD‐induced vitiligo and vitiligo skin have been reported to express IL‐17B, IL‐17D, or IL‐17F rather than IL‐17A [41]. Mast cells play a significant role not only in allergies but also in autoimmune diseases [42]. Therefore, in RD‐induced vitiligo, such cells may be involved in autoimmunity against melanocytes through mechanisms such as T cell mobilization and inflammatory cytokine release. Furthermore, mast cell–derived tryptase reduces keratinocyte GPNMB expression, thereby increasing the vulnerability of newly recruited melanocytes [41]. As mentioned above, CD8^+^ T cells are more likely involved than autoantibodies in the autoimmunity of RD‐induced vitiligo. However, the roles of autoreactive CD8^+^ T cells, resident memory T cells, and regulatory T cell dysfunction in the maintenance, de novo formation, and expansion of RD‐induced vitiligo remain unclear. The lack of an in vivo model that maintains, forms new, or expands RD‐induced vitiligo after RD exposure discontinuation significantly precludes our understanding of these factors. In monobenzone application, a mouse model exhibited self‐reactive CD8^+^ T cell induction [43, 44]. The following mechanisms have been postulated: (1) monobenzone metabolites acting as haptens, thereby inactivating tyrosinase; (2) reactive oxygen species induction; (3) promotion of melanosome autophagy and antigen processing/presentation; (4) release of extracellular vesicles containing tyrosinase/MART‐1; (5) dendritic cell activation and cross‐presentation; and (6) rapid priming of melanocyte‐specific CD8^+^ T cells [43]. Depigmentation at distant sites induced by monobenzone has not been observed in Rag1KO mice [44]. Rag1 encodes a protein that is essential for the V(D)J recombination of immunoglobulin and T‐cell receptor genes, a process that is necessary for the maturation of T and B cells [45]. Deficiency of this protein inhibits differentiation into mature T cells and B cells. This suggests that T or B lymphocytes are involved in depigmentation at non‐exposed sites. Accordingly, in monobenzone, self‐reactive CD8^+^ T cells may contribute to de novo formation and expansion at the lesion site.

### 3.2 Lack of Melanocyte Stem Cells

Stress has been associated with a depletion of melanocyte stem cells [46]. Patients suffering from RD‐induced vitiligo experience a certain degree of decline in their overall quality of life [47, 48]. The observed lack of re‐pigmentation in patients with RD‐induced vitiligo could be explained by melanocyte stem cell depletion. Two reports have co‐stained the hair follicle bulge region, where melanocyte stem cells reside, using FZD4 and MITF, which are markers for melanocyte stem cells or immature melanocytes. A study revealed the presence of stem cells independent of vitiligo severity [49], while another study reported stem cell reduction within the lesions compared with that in the surrounding skin [50]. Notably, patients predominantly affected by completely depigmented patches or those with extensive vitiligo coverage detected no stem cells. Consequently, the existence of melanocyte stem cells remains debatable. Noninvasive methods should be established to confirm the presence and extent of melanocyte stem cells, given their significant impact on future therapeutic strategies.

## 4 Conclusion

This review summarizes the latest insights into the pathogenesis of CL and CIV, focusing on RD. CL/CIV is characterized by the primary loss of mature melanocytes resulting from exposure to certain chemicals. However, the process of melanocyte loss is multifaceted, involving a complex interplay of inducing and suppressing mechanisms. This dynamic interplay contributes to significant individual variability, but numerous aspects of this process are still poorly understood. Specifically, CIV is characterized by chemical exposure as its trigger, followed by numerous pathophysiological features similar to vitiligo. Indeed, CIV and vitiligo share many common elements, particularly autoimmune mechanisms, melanocyte stem cell depletion, and melanocyte niche alterations. However, considering the markedly limited cases relative to vitiligo, the unique pathophysiological aspects of CIV remain unclear. Given that the triggering factors for vitiligo are still largely unknown, CIV investigation may facilitate a deeper understanding of the underlying pathogenesis of vitiligo. A limitation of this review is that clinically distinguishing between CL and CIV is difficult, with a considerable amount of information missing from each report. Such information included the stage and severity of the disease and whether white patches had appeared on unexposed areas. Thus, precisely linking the patient's condition to its mechanism is challenging. Future research connecting clinical information that can distinctly differentiate CL from CIV is warranted. Additionally, in contrast to CL, numerous aspects of CIV pathogenesis remain to be clarified; further research is needed to facilitate the development of effective therapeutic interventions.

## Conflicts of Interest

The authors declare no conflicts of interest.

## Data Availability

Data sharing not applicable to this article as no datasets were generated or analysed during the current study.
